# From smartphone to EHR: a case report on integrating patient-generated health data

**DOI:** 10.1038/s41746-018-0030-8

**Published:** 2018-06-20

**Authors:** Nicholas Genes, Samantha Violante, Christine Cetrangol, Linda Rogers, Eric E. Schadt, Yu-Feng Yvonne Chan

**Affiliations:** 1 Department of Genetics and Genomic Sciences, Icahn School of Medicine at Mount Sinai , New York, NY USA; 2 Department of Emergency Medicine, Icahn School of Medicine at Mount Sinai, New York, NY USA; 3 Sema4, a Mount Sinai Venture, Stamford, Connecticut USA; 4 Epic Applications, Information Technology Department, Mount Sinai Health System, New York, NY USA; 5 Mount Sinai – National Jewish Health Respiratory Institute, New York, NY USA; 6 Center for Digital Health, Icahn Institute of Genomics and Multiscale Biology, Icahn School of Medicine at Mount Sinai, New York, NY USA

**Keywords:** Information technology, Predictive markers, Data integration

## Abstract

Patient-generated health data (PGHD), collected from mobile apps and devices, represents an opportunity for remote patient monitoring and timely interventions to prevent acute exacerbations of chronic illness—if data are seen and shared by care teams. This case report describes the technical aspects of integrating data from a popular smartphone platform to a commonly used EHR vendor and explores the challenges and potential of this approach for disease management. Consented subjects using the Asthma Health app (built on Apple’s ResearchKit platform) were able to share data on inhaler usage and peak expiratory flow rate (PEFR) with a local pulmonologist who ordered this data on Epic’s EHR. For users who had installed and activated Epic’s patient portal (MyChart) on their iPhone and enabled sharing of health data between apps via HealthKit, the pulmonologist could review PGHD and, if necessary, make recommendations. Four patients agreed to share data with their pulmonologist, though only two patients submitted more than one data point across the 4.5-month trial period. One of these patients submitted 101 PEFR readings across 65 days; another submitted 24 PEFR and inhaler usage readings across 66 days. PEFR for both patients fell within predefined physiologic parameters, except once where a low threshold notification was sent to the pulmonologist, who responded with a telephone discussion and new e-prescription to address symptoms. This research describes the technical considerations and implementation challenges of using commonly available frameworks for sharing PGHD, for the purpose of remote monitoring to support timely care interventions.

## mHealth: Smartphone app syncs with health record to improve asthma care

Patients with asthma who record inhaler usage and lung function scores with a smartphone app and transmit the data to an electronic health record (EHR) can get timelier care and prescription adjustments from their doctors. A team led by Yvonne Chan and Nicholas Genes from the Icahn School of Medicine at Mount Sinai in New York, NY, USA explored the feasibility of having patients self-report health data on an iPhone app called Asthma Health and then share the information with their pulmonologists via an EHR patient portal app. Four patients took part in the study, but only two really engaged in the platform. Those patients submitted multiple measures of peak expiratory flow rate per week. In one instance, the measure triggered a pulmonologist to call the patient and prescribe new allergy medications.

## Introduction

mHealth apps and wearable health devices have become increasingly popular among consumers, providing a convenient method of tracking and monitoring fitness, diet, and sleep.^1^ Frequent sampling of objective data such as heart rate and rhythm,^2^ blood pressure,^3^ blood glucose,^4,5^ oxygen saturation,^6^ and activity^7^ for research or clinical care outside of periodic health-care visits is now feasible, and in some cases smartphone apps and related devices have earned US Food and Drug Administration approval and use in clinical workflows.^8^ Moreover, subjective data including standardized surveys or other questionnaires assessing symptoms and other patient-reported outcomes can also be efficiently captured. The ability to collect, summarize, and integrate this data into electronic medical records may facilitate research and clinical care by providing a more nuanced and accurate assessment of health status outside of the typical brief clinical visit or research visit.^9–11^

In 2014, Apple launched HealthKit, a common framework for developers to share patient-generated health data (PGHD) among apps, services, and providers.^12^ ResearchKit, launched in 2015, is an open source framework that gives investigators the infrastructure to develop mobile applications for research via mobile phones. ResearchKit leverages the power of the iPhone’s sensors and connection to third-party devices that monitor activity and other health variables via HealthKit. To support data collection, ResearchKit integrates with HealthKit so that device fitness and health data can be aggregated. Once available in HealthKit, these data can be utilized by other apps and with permission sent to electronic health records (EHRs). Over 1500 apps that make use of HealthKit have been developed, and the types of data collected continues to expand—capturing step counts, body measurements, vital signs, exercise patterns, nutrition, reproductive health, sleep, and more.^13^ This PGHD can serve to supplement clinical data to develop a longitudinal profile of patient health that may ultimately improve management of chronic disease and promote care coordination.

Investigators at Icahn School of Medicine at Mount Sinai along with partners (Apple and Sage Bionetworks) launched the Asthma Health App, a ResearchKit app in which participants could consent to and participate in a research study remotely. Patients were able to securely transmit prospective longitudinal survey data including asthma symptoms, medication use, and other details such as asthma triggers, activity, and location directly to secure study databases.^14^ The app additionally helped facilitate patient education and self-monitoring, informed users of local air quality, and promoted adherence to treatment plans. The app also allowed the option for participants to enter peak expiratory flow rate (PEFR) as captured by the patient’s own home peak flow meter into the app for integration with other study data. As rescue medication use and PEFR are widely accepted as useful variables for clinical monitoring of asthma, we piloted the feasibility of integrating medication and PEFR data collected for research purposes from the Asthma Mobile Health Study into our local clinical EHR, Epic Systems (Verona, WI), for potential use in clinical care.

### Objectives

This case report evaluates the technical aspects of data integration as well as exploring the implications for continuous, patient-generated data collection paired with EHRs for disease management.

## Results

**Fig. 1:**
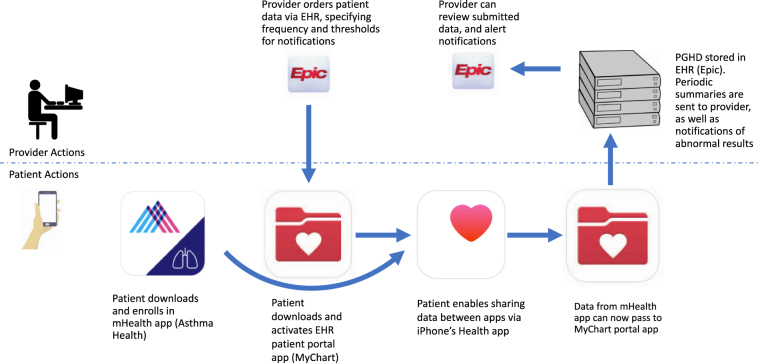
After patient/provider discussion and consent, both must act to enable PGHD collected in the patient’s mHealth app to appear in the provider’s EHR

**Fig. 2:**
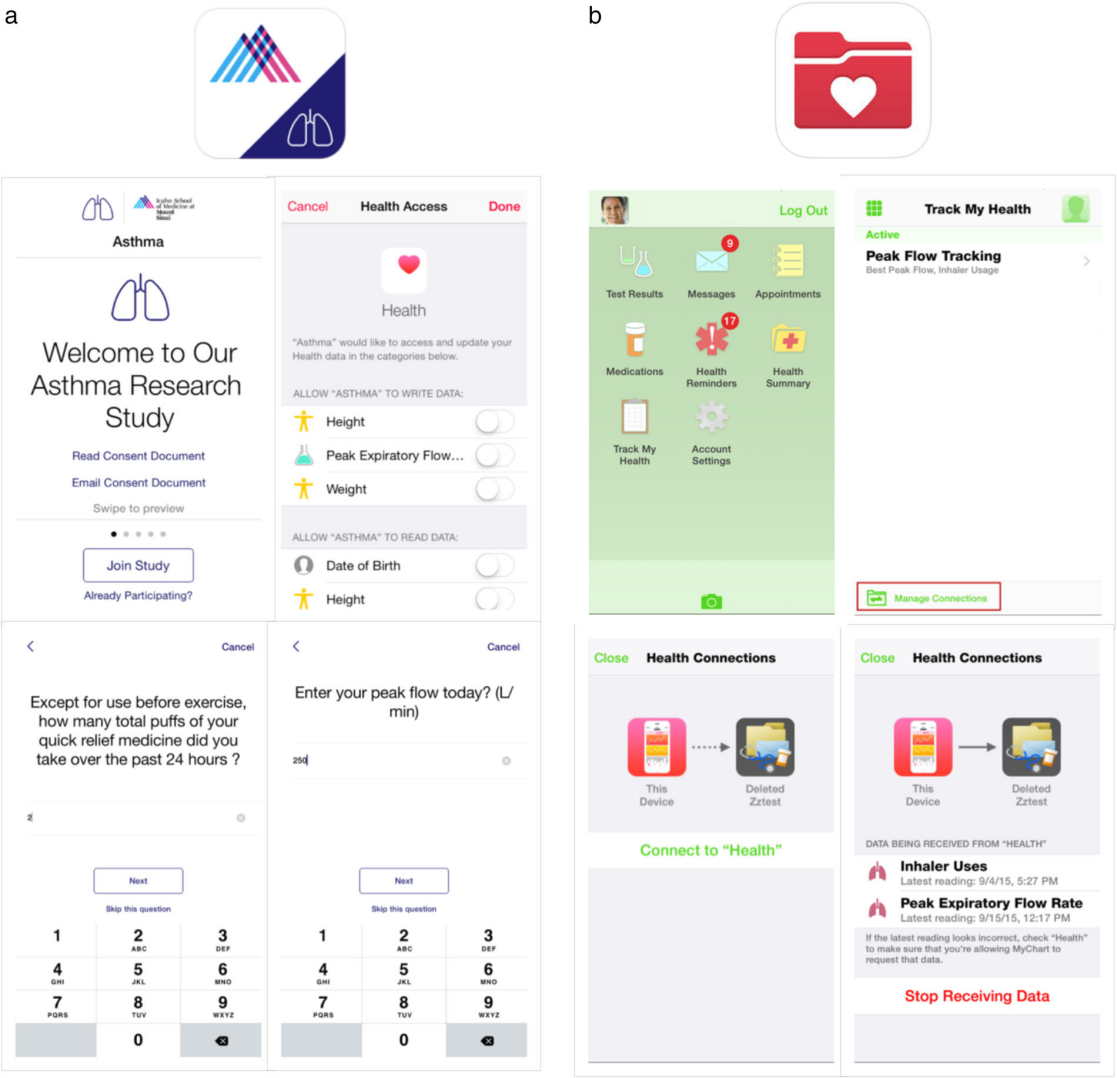
Patient views from the Asthma Health app and Epic’s MyChart app to enroll and enable sharing of PGHD. **a** After downloading the Asthma Health app, the patient enrolls and follows prompts to allow sharing of data with the iPhone’s Health App. Then patient is regularly prompted to enter inhaler use and PEFR. **b** After downloading and activating MyChart, the patient sees a message about a provider's peak flow tracking order and is prompted to connect data from the iPhone’s Health app to MyChart

In early 2016, a pulmonologist (L.R.) placed orders for inhaler usage and PEFR data in Epic for four patients with asthma under her clinical care who had consented and enrolled in the Asthma Mobile Health Study. They were instructed how to activate Epic’s MyChart app on their iPhones and agreed to forward their peak flow and medication use data being collected for research purposes in the Asthma Health Study to their clinical chart in their EHR for use in their clinical care (see Figs. 1 and 2).

Two patients submitted just a single PEFR data point apiece through the course of this 4.5-month trial, and for one of those patients, the data point was not physiologically valid (“0”). Another patient, shortly after the pulmonologist’s order for data, submitted 101 PEFR data points across a span of 113 days (on many days, 2 PEFR values were collected—the patient submitted data on 65 distinct days). The lowest PEFR was 250 L/min (62.5% of this patient’s personal best), the highest was 400 L/min, and the average PEFR was 310 L/min (77.5% of personal best and 70.5% of predicted best, based on age and gender). On one occasion, inhaler usage was noted. The fourth patient, beginning several months after the pilot period began, submitted 24 PEFR data points across 66 days (never more than one PEFR per day) but also indicated inhaler usage on all 24 days where PEF data wēre submitted. The lowest PEFR (aside from one collected value of 0) was 300 L/min (66% of personal best), the highest was 450 L/min, and the average PEF was 376 L/min (85.5% of personal best, which was approximately equivalent to predicted best, based on age and gender).

Altogether during the 142 days when PGHD was monitored for this project, 127 PEFs were captured in the EHRs, and 25 inhaler usage episodes were captured in the EHRs. One PEFR fell outside the predefined threshold, triggering an EHR alert for the pulmonologist (L.R.).

**Fig. 3:**
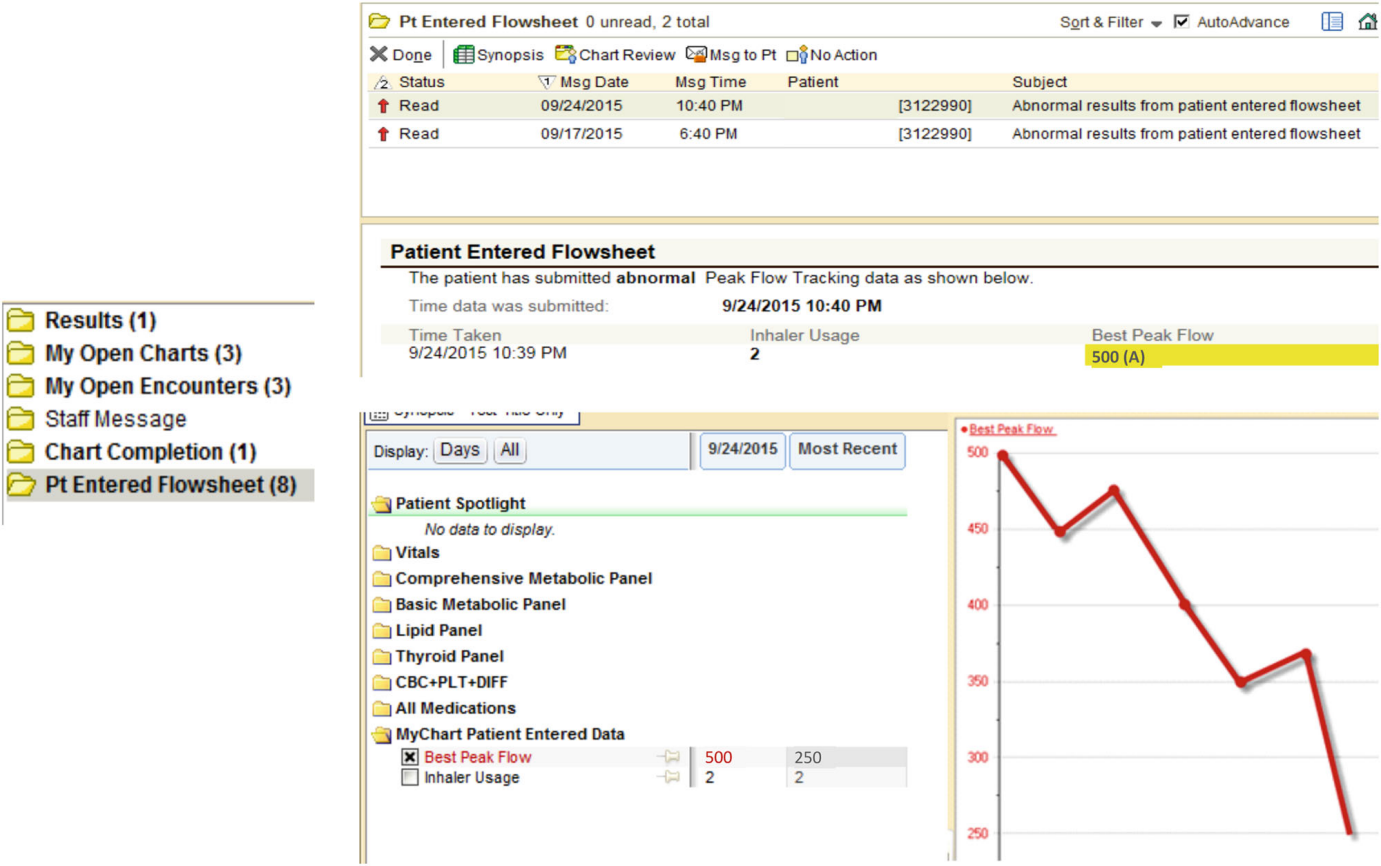
Provider's view in Epic’s Inbasket, when reviewing patient peak expiratory flow rates (this is simulated data from a virtual patient)

Upon reviewing collected PEFR data for one patient, L.R. noted a drop in peak flow <60% of personal best, an accepted threshold for triggering a clinical intervention (see Fig. 3) and so contacted the patient over the phone to learn if the patient had an explanation. The patient-reported allergy medications had run out, and L.R. e-prescribed new medications to the patient. No clinic visits were arranged or postponed as a result of the data collection, and no emergency department visits were noted among the four patients during the observation period.

## Discussion

While the transmission of PGHD stored in HealthKit with an EHR has been previously described,^15^ and custom integrations have been reported,^4,16^ case reports assessing the technical aspects of such an integration, for clinical purposes, remain rare. The effort demonstrated a bidirectional flow of information between the patient and clinician (the clinician placing the order for data, and the patient supplying the ordered data), using the methods commonly available in smartphone apps and a leading EHR. PGHD allowed the clinician to provide feedback to the patient on daily asthma control and use of rescue medications. This information has the potential to be followed over time and improve chronic disease management. Chronic care management (CCM) codes have been developed to incentivize providers to analyze PGHD in select populations^17^; now iPhone apps that make use of HealthKit can fall under this program. The ability to receive notifications—specifically, warnings when a patient’s peak flow reading (or any predetermined parameter of clinical interest) falls outside an established range—could allow for early interventions, such as a telephone call to check up on the patient, or adjustment of prescriptions. Integrated notifications to an EHR, prompting more frequent and directed clinical communications, may lead to improved clinical care.

Although the potential and impact of this integration is exciting, challenges exist in implementation and in incorporating a PGHD in current workflows. Technical hurdles included the requirement for participants to have an iPhone 5 or newer model due to ResearchKit requirements and excluded users of Android or other such devices. In terms of training, pulmonologist L.R. made time in her schedule to learn the setup process of initiating new orders for patients. Once learned, the setup process for the clinician was simple and easy to initiate, though some care was taken in recruiting patients for the pilot, given the lengthy setup process for patients (installing and activating MyChart, consenting to the Asthma Health app, and permitting data sharing). In future, dedicated staff for training and recruitment of participants may facilitate the process. Dedicated support and distribution of clear instructions were effective in counterbalancing the setup process. Several major EHR vendors have embraced FHIR (Fast Healthcare Interoperability Resources) which, among other things, aim to simplify the integration process of approved mHealth apps into EHRs, facilitating the sharing of PGHD.^18^

As we approached only patients who were participating in the Asthma Mobile Health Study locally in NY, who saw a provider who was one of the study investigators [L.R.], only four patients were enrolled. As this was a pilot, we wanted to ensure that the process worked correctly prior to making it available to other providers. L.R. approached these four patients who were known to be participating in the study and had previously expressed interest in using study data for clinical purposes.

In the context of this study, patients entered their peak flow and medication use data into the Asthma Health App for the purposes of an existing research study (other survey questions in the app were focused on symptoms and perceived triggers, and were not transmitted to the clinicians’ EHR). The workflow between the app and Epic was developed to enable the passive collection of clinical data, originally intended for research purposes (specifically to enable the passive collection of data from activity monitors and other medical devices, including data from Bluetooth-enabled peak flow meters and inhalers currently under development), provided the research participant opted to share that data. Otherwise, a patient’s HealthKit data could only be used for personal purposes, or shared with a provider if desired, by connecting with MyChart. To then transmit research that data to a clinician, there would need to be communication between the app’s underlying ResearchKit framework, HealthKit, and the MyChart app. Although this setup is somewhat complicated, it is what allowed automatic transmission of passive collection of research data and then forwarding it directly to the patient EHR for clinical use and allowed for the patient or research participant to separately control who has access to data.

Though newer methods like Apple’s CareKit framework and the FHIR protocol may facilitate simpler ways of sharing data, at the time of this study, this use of ResearchKit, HealthKit, and MyChart/Epic were designed to function in this manner. This interaction between three systems did create some complexity and some challenges with training and setup—for both our research team and patients participating in the integration. For purely clinical use of data, a workflow could have been developed between HealthKit and the MyChart app without involving ResearchKit.

Although the system of integration in this case did add some complexity, this workflow did lay the foundation for use of Bluetooth-equipped devices to automatically upload data of inhaler use and peak flows automatically into either a research app or a clinical app that could then be linked to an EHR. Data from Bluetooth devices could be thus voluntarily shared by the participant for both research purposes and clinical purposes (these types of external devices were not used in the current study, however).

Even when technical hurdles are overcome, however, processing and acting on incoming PGHD need to be addressed. Providers and care teams will have to develop workflows to meet the challenge of reviewing data and following up on patients with concerning trends and outlier values.^11^ Reimbursement for remote monitoring in the US (through CCM billing and Merit-based Initiative Payment System) may help facilitate the hiring of dedicated staff or protecting the provider’s time necessary to review PGHD. However, not all patients with PGHD qualify for CCM, and incorporating a CCM process into clinical practice still typically requires investment, training, and the help of consultants.

Despite the lengthy integration process for patients, those approached seemed motivated to join this effort. The benefits of patients participating in any EHR integration can support improved care coordination, through enhancing patient engagement and improved decision making for all stakeholders.^11^ The graphic function in Epic allows visualization of objective measurements of lung function that patients can map along with frequency of rescue medicine use to understand trends in their symptoms and lung function. Data that are automatically sent to providers can help providers to assess peak flow variability without cumbersome paper diaries and hand calculation of percent peak flow variability that is difficult and time consuming to perform in practice. Graphic display of data trends provides a sense of asthma control of the monitoring period visually and without a need to enter hand-collected data into a separate analytic program.

Limitations include that many patients do not adhere with regular continual peak flow monitoring or adhere for a period and then lose interest. Clinical scenarios where one could envision focused implementation and strong emphasis on serial peak flow monitoring include patients with severe asthma, particularly exacerbation-prone patients or patients with a history of intubation/near fatal asthma. This tool could also be useful in diagnosis of work associated asthma where trends in disease can be mapped to work days and work-related exposure. Lastly, this would be extremely useful in patients with poor perception of airflow limitation for whom changes in therapy would need to be made based on measured peak flow values even in the absence of symptoms.

In summary, while mobile app and EHR data transmission is still novel, this is one of the first ResearchKit apps where clinical data collected via smartphone app has been transmitted directly to an EHR. Mobile health applications are a useful vehicle for engaging patients and can lead to improved care coordination. There are a range of clinical and financial benefits from enabling EHR integration with mobile health apps.^16^ This cost-effective method of generated patient health data can catalyze the use of big data to enhance medical research.^19^ Consumer technology companies like Apple recognize the changing landscape of health care and have opened the doors to greater patient data collection with the launch of HealthKit and ResearchKit and, most recently, CareKit, a framework that provides patients the ability to manage their own medical conditions, track care plans, and monitor symptoms and medications, as well as securely share this information with doctors, nurses, or family members.^20^ The collection of HealthKit data and its integration with EHR companies, such as Epic and Cerner, has tremendous potential to close the gap on data obtained through intermittent visits with health-care providers in traditional health-care settings.^11^ With continued advancement in technology and potential for EMR integration, there is enthusiasm and momentum to continue to advance medical research and improve health outcomes.

## Methods

### System design—HealthKit

During the Asthma Mobile Health Study, Asthma Health app users shared their survey responses regarding PEFR readings and rescue medication use data with Sage Bionetworks servers for analysis by investigators. Study participants are also given the opportunity to share and import their iPhone’s HealthKit data with investigators, such as step count (as determined by the iPhone device or third-party devices) and heart rate (measured by third-party devices) as of version 1.1 of the Asthma Health app. If a user permits, data can be seamlessly shared among a user’s apps, as all ResearchKit and HealthKit fields were encoded using HL7 standards. During the course of our study, we released an upgraded version of the Asthma Health app that asked users for permissions to write data into HealthKit and modified app-administered surveys so that selected survey values (specifically, PEFR and rescue medication usage) could be written into each participating user’s HealthKit repository. The Asthma Mobile Health Study was approved by the Mount Sinai Program for the Protection of Human Subjects (#14-00964), and these methods were performed in accordance with relevant regulations and guidelines.

### System design−Epic

Epic Systems was among the first EHR vendors to leverage HealthKit for collecting and transmitting PGHD to providers through their iPhone app, called MyChart.^21^ To transfer HealthKit data to an EHR, a number of steps are required (see Fig. 1): (1) A provider at the site of clinical care must “order” their patient’s data; (2) a patient must use an eligible iPhone and have the MyChart app installed and activated; and (3) an app or device (in this case the Asthma Health app) collecting data (in this case PEFR and inhaler usage) must be configured to give permission for the data to be shared with Health Kit and MyChart in order for data to flow from the patient’s phone to the EHR. Details of design and implementation of this process are outlined below.

### Clinical information and workflows

**Fig. 4:**
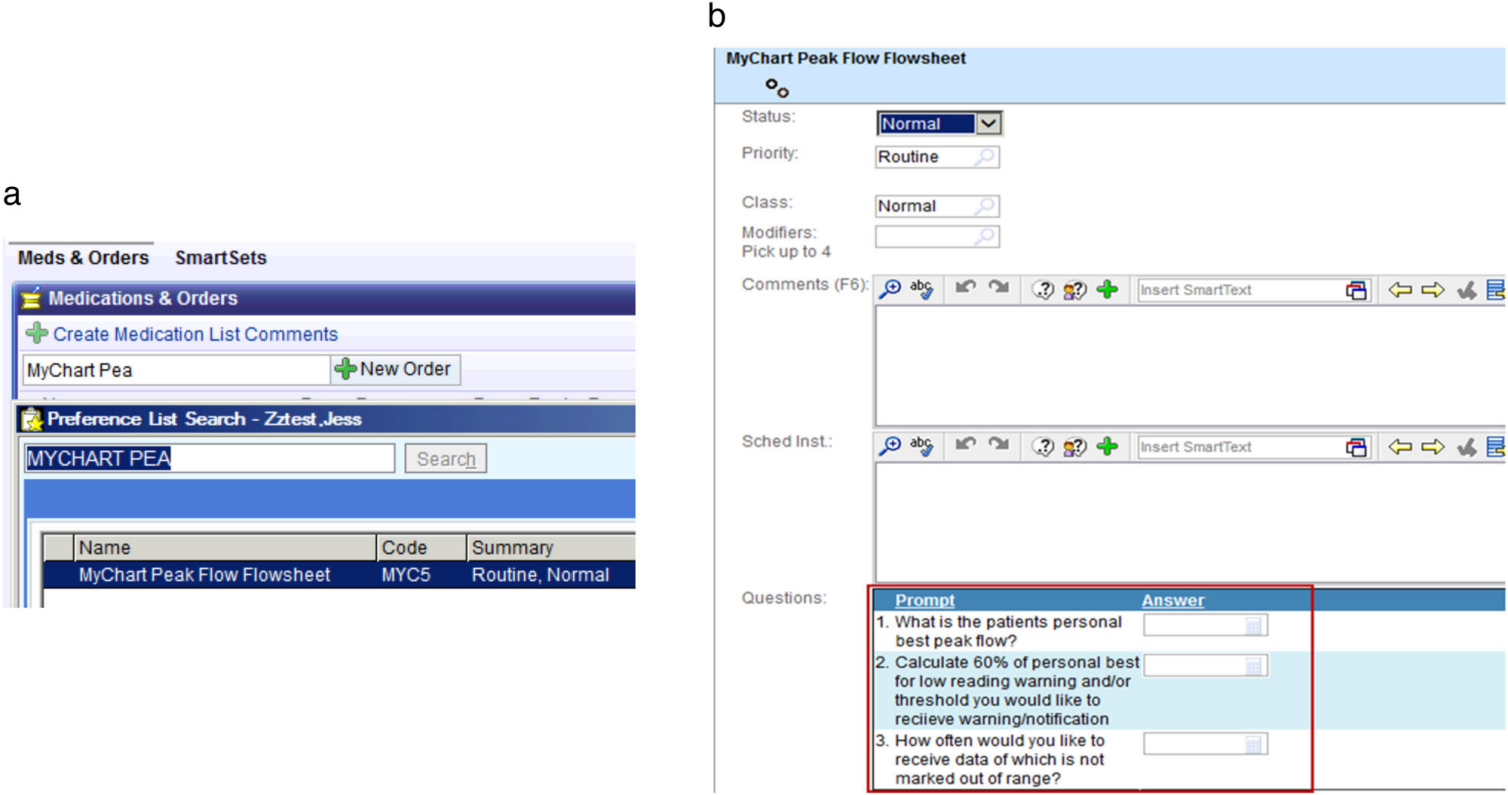
The provider’s view in the Epic EHR. **a** The provider’s Epic view, when searching to order a patient’s peak expiratory flow rates. For data to flow into an Epic chart (specifically, a flowsheet), it must come from a patient’s MyChart app. **b** The provider’s Epic view when specifying notification settings for a patient’s peak expiratory flow rate data, including frequency and thresholds for notification

In order to allow PEFR data and medication usage to flow from an user’s iPhone to the Epic EHR, an order was designed for providers to initiate PEFT and inhaler usage tracking, in Epic, of patients in that provider’s practice (see Fig. 4). Patients cannot transmit data to providers without an explicit order; in the course of placing this order, the provider may enter parameters for notifications. The provider can specify the patient’s expected personal best PEFR, a range of normal values indicating stable asthma (for instance, 80–100% of personal best PEFR) to low values that are 60% or less of the patient’s personal best PEFR (a level that may indicate a need for a clinical intervention or change in asthma therapy). The provider can also specify the frequency at which they would like to receive data, from daily notifications to monthly graphs summarizing PEFR data.

### Patients’ enabling data sharing

To use a ResearchKit app such as Asthma Health, study participants needed to have an iPhone 5 or later or an iPod Touch (purchased 2012 or later) running iOS 8 or later. Eligibility criteria for the Asthma Health Study included physician-diagnosed asthma, US residency, and age of at least 18 years. Provided the study criteria were met, the user was able to download the Asthma Health App and consent to the study through an e-consent process.^14^ Once consented, the user was prompted to grant the Asthma Health App access to pull in general health information from the HealthKit (See Fig. 3). At this point, a toggle switch also appears in the app to enable, if activated, reading and writing PEFRs to the Asthma Health App. If the study participant was already enrolled in the study, the participant could confirm that this feature was turned on by navigating directly to the Health App on their iPhone to view the source of the data (in this case, the Asthma Health app).

A similar connection between HealthKit and MyChart was needed to allow peak flow information to arrive in MyChart (see Fig. 3). The study participant is required to first log into MyChart (the username and password needed to activate MyChart is provided at the site of the heath-care visit), select “Track My Health”, and then “Manage Connections”. Within the Health Connections screen, the user must select Connect to Health to enable the data connection from MyChart to HealthKit. There is an option for the user to disable this connection at any time.

In order to study the impact of clinically sharing this PGHD, instructions to activate the data flow were prepared for both patients and providers (see Supplemental Figs. 1 and 2). There were multiple test runs conducted between Mount Sinai’s Information Technology group and Respiratory Institute and the Asthma Mobile Health Study/Icahn Institute for Genomics and Multiscale Biology’s Digital Health team.

### Data availability

The datasets generated during the current study are available from the corresponding authors, on reasonable request.

## Electronic supplementary material

Supplementary Figures 1 and 2

## Electronic supplementary material

**Supplementary information** accompanies the paper on the *npj Digital Medicine* website (10.1038/s41746-018-0030-8).
