# Supplementary material for: From smartphone to EHR: a case report on integrating patient-generated health data
Source: NPJ Digit Med. 2018 Jun 20;1:23. doi: 10.1038/s41746-018-0030-8 (PMC6550195; doi:10.1038/s41746-018-0030-8)
Supplement: Supplementary file 1 — Supplementary Figures 1 and 2 [file 41746_2018_30_MOESM1_ESM.docx]

**Supplementary Figure 1.**

**Viewing and Working with Asthma Health app data, in Epic: Instructions for Providers.**

**P**roviders can receive summaries of patients’ peak flow readings, as well as alerts if a patient’s peak flow falls below a threshold. The frequency of reports and alerts can be determined by a patient’s provider, in Epic.

**Requirements for use:**

1. Patients must have an iPhone 5 or later, an iPod Touch (purchased in 2012 or later), and be running iOS 8 or later.
2. Patients must also have asthma, live in the US, and be age 18 or older.
3. The patient needs MyChart access and the MyChart app on their iOS device.
4. Confirm the patient has downloaded the Asthma Health App, consented, and enabled data sharing with HealthKit (done at startup, or enabled later through the Health app’s Sources tab).
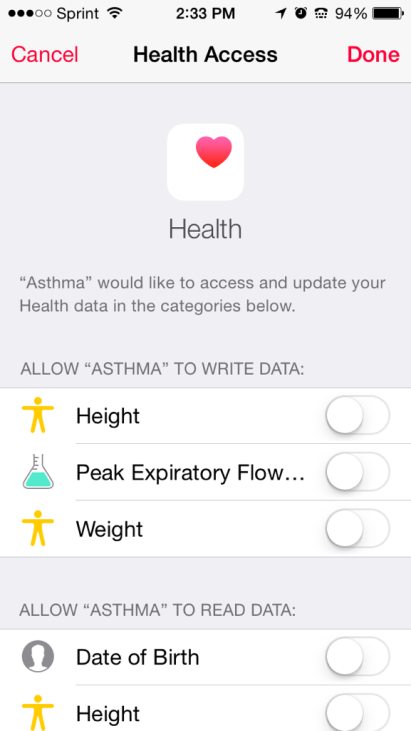


**To obtain new Peak Flow data and enable notifications:**

1. Select New Order in Epic, in the search box type “MyChart Peak Flow”
2. Complete the order form by filling in the following *mandatory* fields:
   - 1. What is the patient’s personal best peak flow?

This is necessary to calculate deviations from ideal PEFR.

- - 1. Calculate 60% of personal best for low reading warnings and/or threshold you would like to receive warning notifications?
    2. How often would you like to receive data of which is not marked out of range?

Data can be received every 1 to 30 days.

Once an order is placed:

1. You will receive notifications in your Epic InBasket when there is abnormal data to review.
2. If abnormal readings require intervention, the provider or support staff should reach out to the patient directly. The patient will not receive any notification from Epic.
3. Routine data can be trended using Synopsis tool from the InBasket.

For questions or additional support please contact the Help Desk (x4HELP).

**Supplementary Figure 2.**

**Instructions for Patients to track asthma symptoms and triggers, in the Asthma Health App**

The Asthma Health Dashboard includes a concise summary of your asthma symptoms over time, organized in a way that can be easily shared with your doctor. This Dashboard helps transform the data you share, into a tool that you and your doctor can use to help guide medical decisions and provide you with better care.

Utilizing the Asthma Health App and Apple’s Health app will enable the transfer of peak flow information to your physician. Your doctor can receive a summary of your peak flow readings, as well as alerts if your peak flow falls below a threshold. The frequency of reports and alerts are determined by your doctor.

**Requirements for use:**

1. You must have an iPhone 5 or later, an iPod Touch (purchased in 2012 or later), and be running iOS 8 or later.
2. You must also have asthma, live in the US, and be age 18 or older.
3. The MyChart app needs to be downloaded to your iOS device from the Apple App Store. Your doctor will provide a username and password to access MyChart.

**Step 1: Download the Asthma Health App from the Apple App Store:**

- - - 1. Download Asthma Health by Mount Sinai from the Apple App Store
      2. Follow the eConsent process in the app and consent to the study
      3. Once consented, you will be prompted to grant the Asthma Health App access to pull in general health information from the Apple Health app (Health Kit). To enable this information to be available in Asthma Health switch the toggle to green. PLEASE NOTE: In order for your doctor to receive summaries of your peak flow readings, the peak flow option must be turned on. Once your selections are made, select Allow in the upper right and continue with the user registration.


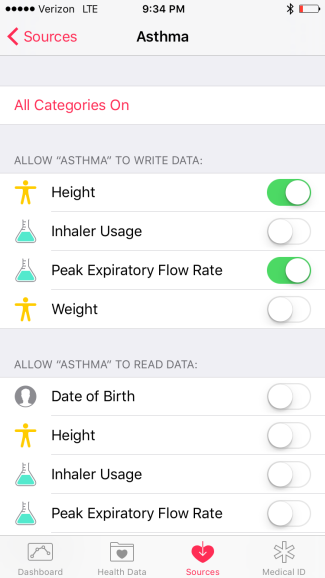


In this example, Peak Flow and Height is being read into the Asthma Health App

**Confirmation Step: If you’re already enrolled in the Asthma Health study, and want to confirm Peak Expiratory Flow Rate is enabled in Apple Health app:**

1. Open Apple’s Health app
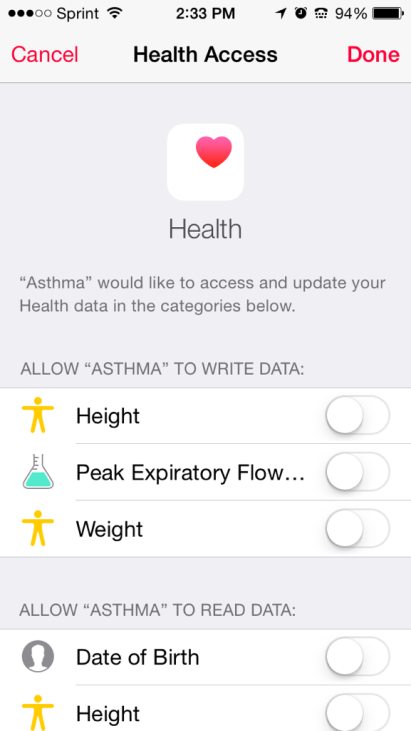

2. Select “Sources” along the bottom
3. Select Asthma
4. Locate “Peak Expiratory Flow Rate” and turn the toggle to display green, to enable your doctor to receive summaries of your peak flow readings (toggle should be green for “Allowing Asthma to write data” and “Allow Asthma to read data”) (see image above in step 1)

**Step 2:** Enable Data Sharing in MyChart:

1. Open and log in to MyChart App, Select Track My Health (Image 1).
2. Within the “Track My Health” screen, select Manage Connections (Image 2)
3. Within the “Health Connections” screen, select Connect to “Health” (Image 3).

Click here

1. A final screen will appear, confirming the transfer of data to MyChart (Image 4). Select Close (upper left).


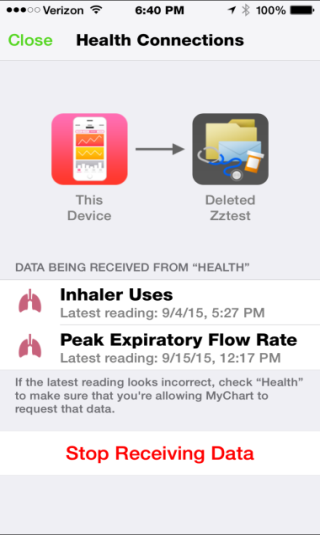

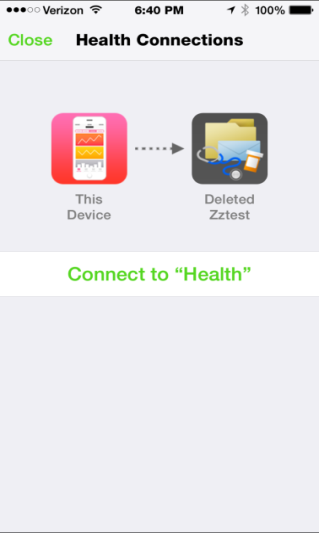

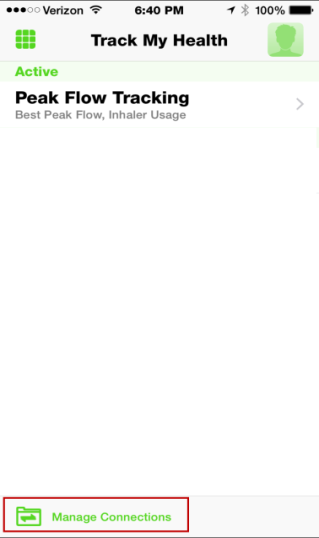

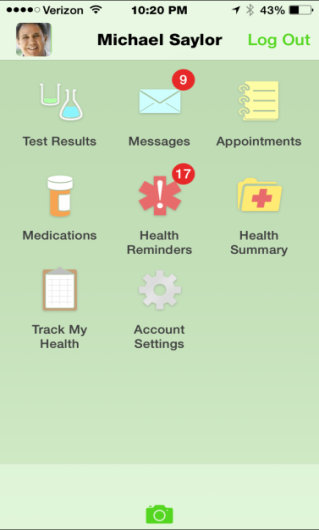


Image 1

Image 3

Image 4

Image 2

Click here

Click here

Select Track My Health

**What happens next?**

As a participant of the Asthma Health App, you will complete a short daily survey to assist in tracking triggers, peak flow and adherence to medication. This information can be shared with your physician directly. This information will also be available to your doctor in your electronic health record. If your peak flow falls below what your doctors specifies as an abnormal reading, an alert message will be sent to providers for observation or intervention.

For any questions regarding the Asthma Health App please contact: [asthmamobilehealth@mssm.edu](mailto:asthmamobilehealth@mssm.edu).
